# Biomechanical drivers of the evolution of butterflies and moths with a coilable proboscis

**DOI:** 10.1098/rspb.2024.0903

**Published:** 2024-11-27

**Authors:** Alexandre V. Palaoro, Daria Monaenkova, Charles E. Beard, Peter H. Adler, Konstantin G. Kornev

**Affiliations:** ^1^ Department of Materials Science and Engineering, Clemson University, Clemson, SC 29634, USA; ^2^ Departamento de Zoologia, Universidade Federal do Paraná, Curitiba, Paraná 82590-300, Brazil; ^3^ Department of Plant and Environmental Sciences, Clemson University, Clemson, SC 29634, USA; ^4^ Department of Biological Sciences, Clemson University, Clemson, SC 29634, USA

**Keywords:** evolution, Lepidoptera, proboscis, hawkmoth, feeding

## Abstract

Current biomechanical models suggest that butterflies and moths use their proboscis as a drinking straw pulling nectar as a continuous liquid column. Our analyses revealed an alternative mode for fluid uptake: drinking bubble trains that help defeat drag. We combined X-ray phase-contrast imaging, optical video microscopy, micro-computed tomography, phylogenetic models of evolution and fluid mechanics models of bubble-train formation to understand the biomechanics of butterfly and moth feeding. Our models suggest that the bubble-train mechanism appeared in the early evolution of butterflies and moths with a proboscis long enough to coil. We propose that, in addition to the ability to drink a continuous column of fluid from pools, the ability to exploit fluid films by capitalizing on bubble trains would have expanded the range of available food sources, facilitating diversification of Lepidoptera.

## 1. Introduction

The ability of butterflies and moths (Lepidoptera) to retrieve fluids deep in flowers has long fascinated naturalists. Hawkmoths with proboscises more than 20 cm long acquire nectar from long-spurred flowers where nectar viscosity varies as the sugar content changes with temperature, moisture availability and flower species [1].

Hawkmoths typically hover while drinking from flowers. Paradoxically, the time spent by a hawkmoth acquiring nectar from long spurs is shorter [2] than the time needed to form a continuous liquid column in the proboscis. The long-tongued hawkmoth, *Cocytius antaeus*, typically spends less than 1 s feeding from the ghost orchid, *Dendrophylax lindenii* [3]. The biomechanics of feeding by long-tongued hawkmoths thus remains enigmatic [4].

**Figure 1.**
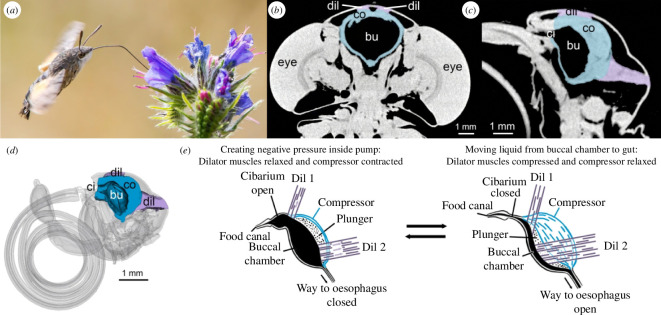
Sucking pump in Lepidoptera. (*a*) *Macroglossum stellatarum* hovering with its proboscis uncoiled to reach the floral nectar (photo by James and Dawn Langiewicz). (*b*) Frontal micro-computed tomography (µCT) slice of the head of *Manduca sexta*, showing the buccal chamber (bu), compressor muscles (co, in blue) and dilator muscles that attach to the top of the plunger (dil, in purple). (*c*) Lateral view of the head of *M. sexta*, showing dilator muscles (dil) that attach to the compressor muscles and plunger (co, in blue), the buccal chamber (bu) and smaller cibarium (ci). (*d*) Three-dimensional reconstruction of the head of *M. sexta* sectioned parallel to the plane of the coiled proboscis and reconstructed with three-dimensional microtomography software. (*e*) Schematics illustrating the action of compressor and dilator muscles that open and close the sucking pump, respectively.

Drinking straw models of fluid uptake assume that hawkmoths *drink* nectar, that is, nectar continuously flows through the proboscis to the sucking pump [5,6]. Thus, to defeat the viscous drag of nectar passing through the proboscis, hawkmoths should create a large pressure differential, requiring large sucking pumps with stronger, more voluminous muscles as proboscis size increases (figure 1; [5,6]). In the alternative model to explain rapid drinking [4], fluid enters the food canal through legular bands so that neither size of the sucking pump nor muscle volume would limit feeding rate. Rather, galeal curvature and permeability of interlegular slits of the proboscis control the rate of fluid uptake [4].

Data on the sucking pump and muscle volume are lacking; therefore, feeding scenarios remain speculative. Quantification of sucking pump characteristics, such as volume of the buccal chamber, dilator muscles and compressor muscles (figure 1) in relation to food canal volume and proboscis length and morphology, is pivotal to understanding lepidopteran feeding. The hawkmoth family Sphingidae, with more than 1460 species and diverse proboscis geometries and lengths, provides a model for comparative analysis.

Having commonly observed bubble-train formation in the proboscis [7,8], we hypothesized that lepidopterans employ alternative feeding modes that do not rely solely on imbibing continuous liquid columns. We, therefore, investigated the biomechanics associated with fluid uptake in Lepidoptera.

We tested the conventional feeding scenario that butterflies and moths with a coiled proboscis (suborder Glossata) pull a continuous liquid column through the proboscis–sucking pump complex. To expose challenges with the drinking-straw scenario of fluid uptake, we investigated the capacity of the sucking pump with respect to the proboscis capacity and proboscis resistance to transporting continuous liquid columns. We integrated micro-computed tomography (µCT), capillary rise experiments and mathematical modelling to evaluate flow dynamics and morphology in 33 species of hawkmoths. To elucidate the evolution of the feeding mechanism, we added synchrotron X-ray imaging of monarch butterflies (*Danaus plexippus*) and µCT scans of six butterfly species. We then synthesized the evidence to explain the evolution of fluid drinking in Lepidoptera. Comparing hawkmoths and butterflies allowed us to suggest a potential evolutionary route for feeding by adult Glossata.

## 2. Volumes of the buccal chamber and muscles, and sucking pump–proboscis capacity and resistance

Anatomical features related to biomechanics of the sucking pump–proboscis complex of adult Lepidoptera have been discussed [5,6,9]. We focus on the most critical structures (figure 1*e*) in operation of the sucking pump. Fluid moving from the proboscis enters the cibarium, which is connected to the larger buccal chamber. The flexible roof (plunger) of the buccal chamber is driven by dilator muscles attached to the head capsule and plunger. Contracting the dilators pulls up the roof of the buccal chamber, creating negative pressure, simultaneously closing the oesophageal valve [10,11]. Negative pressure in the buccal chamber opens the food canal and cibarium, bringing liquid from the proboscis into the chamber [10,11]. At this stage, the muscular energy is mainly spent overcoming viscous drag when fluid moves through the food canal [11]. Compressor muscles close the food canal and cibarium. These muscles run from the dorsum of the buccal chamber to the dorsoventral junction of the pump floor. Contracting these muscles closes the cibarium and opens the oesophagus, allowing the insect to swallow. During this step, muscular energy is spent on overcoming viscous drag in the chamber [11].

Two feeding scenarios are possible. Sipping occurs when the volume of the buccal chamber is less than that of the food canal: the chamber must open and close multiple times to empty the food canal. However, if the volume of the buccal chamber is greater than that of the food canal, the entire food canal volume can be swallowed in one gulp.

**Figure 2.**
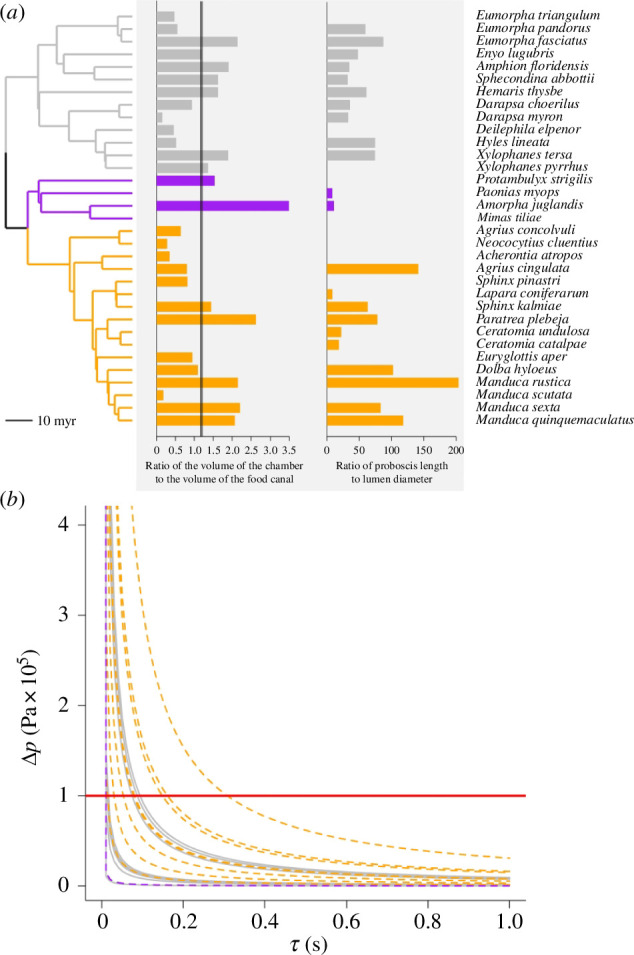
Biometrics of the sucking pump–proboscis complex. (*a*) Hawkmoth phylogeny (far left panel), based on [12] for species in our study. Colours represent different subfamilies of hawkmoths: grey, Macroglossinae; purple, Smerinthinae; and yellow, Sphinginae; black represents the common ancestor of Sphinginae and Smerinthinae. The scale represents the timespan of the phylogeny in millions of years (Myr). The first panel to the right of the phylogeny shows the ratio of the volume of the buccal chamber to the volume of the food canal. The vertical black line denotes the average, showing that the lumen can hold an average of 1.2 times the volume of the food canal. Values above 1 indicate that the volume of the buccal chamber is larger than that of the food canal. Species without a bar have galeae that are often unlinked, which prevented us from calculating the volume of the food canal. The next panel to the right indicates the ratio of proboscis length to its lumen diameter. Species without bars lacked a measurement for lumen diameter. (*b*) Relationship between pressure drop (Δ*p*) and the time to open the buccal chamber (*τ*) calculated using equation (2.1). Colours represent subfamilies as in (*a*). Dashed lines used only to enable visualization of all lines in the plot.

Our µCT scans revealed that the buccal chamber of hawkmoths can hold an average of 1.2 times the volume of the food canal $V_{fc}$ (mean ± s.d. = 1.2 ± 0.7, *n* = 28, one-sample *t*‐test: *t*_27_ = 1.7, *p* = 0.1, figure 2*a*), with considerable variation among species. The species with the largest ratio of buccal chamber $V_{bc}$ to food canal volume $V_{fc}$ were *Paratrea plebeja* ($V_{bc}$ ≈ 2.7$V_{fc}$) and *Amorpha juglandis* ($V_{bc}$ ≈ 3.5$V_{fc}$), whereas the species with the smallest ratios were *Darapsa myron* ($V_{bc}$ ≈ 0.15$V_{fc}$) and *Neococytius cluentius* ($V_{bc}$ ≈ 0.28$V_{fc}$). Thus, while some hawkmoths can drink the entire food canal volume in a single sip, others require multiple sips, suggesting that modes of drinking might be labile across species.

First, we analyse a scenario where the insect sips continuous liquid columns of viscosity η with the rate $Q=\frac{V_{bc}}{\tau }$, where *τ* is the time to open the buccal chamber. By modelling the food canal as a straight cylinder of radius r and length $L_{c}$, the Hagen–Poiseuille equation for pressure drop Δp generated by the sucking pump is


(2.1) $$\Delta p=8\eta \frac{QL_{c}}{\pi r^{4}\;}=8\eta \frac{V_{bc}}{\tau \pi r^{2}\cdot L_{c}\;}\cdot \frac{L_{c}\cdot L_{c}}{r^{2}}=8\eta \left(\frac{V_{bc}}{V_{fc}}\right)\cdot {\left(\frac{L_{c}}{r}\right)}^{2}\cdot \frac{1}{\tau }.$$

The ratio $V_{bc}/V_{fc}$ signifies the importance of proboscis–buccal chamber coupling: in the organism, this pair is connected. Like taking liquid from a large pool with a syringe, for a given time τ, the volume of acquired fluid can be measured in the needle volume. Therefore, capacity $\left(V_{bc}/V_{fc}\right)$ appears as a metric of relative volume of absorbed fluid. We show that $V_{bc}/V_{fc}$ stays within the same order of magnitude but $L_{c}/r$ changes are significant (figure 2*a*). Small changes in τ cause large changes in Δ*p* (figure 2*b*). To compare our results with those for *Manduca sexta* from [13], we used τ = 1s to obtain Δp = 0.014 MPa. Pierce & Hedrick [13] estimated Δp ≈ 0.01 MPa by measuring the flow rate of *M. sexta* drinking from a tube. Thus, equation (2.1) based on measured morphological parameters of the proboscis-buccal chamber pair appears reliable.

The strength of the dilator muscles is reflected in equation (2.1) by the time τ required to open the buccal chamber (electronic supplementary material).

The sucking pump and its muscles had similar conformation across sphingid subfamilies and feeding habits (electronic supplementary material, figure S1). Aside from differences in proboscis length, the main difference across species was in muscle volume. To examine if muscles scale disproportionally to the food canal, we performed a phylogenetic regression between volume of compressors and dilators against volume of the food canal while performing an ancestral state reconstruction of the volume of both muscle sets. We excluded *Ceratomia catalpae* and *Mimas tiliae* from these calculations because their galea do not form a closed food canal.

**Figure 3.**
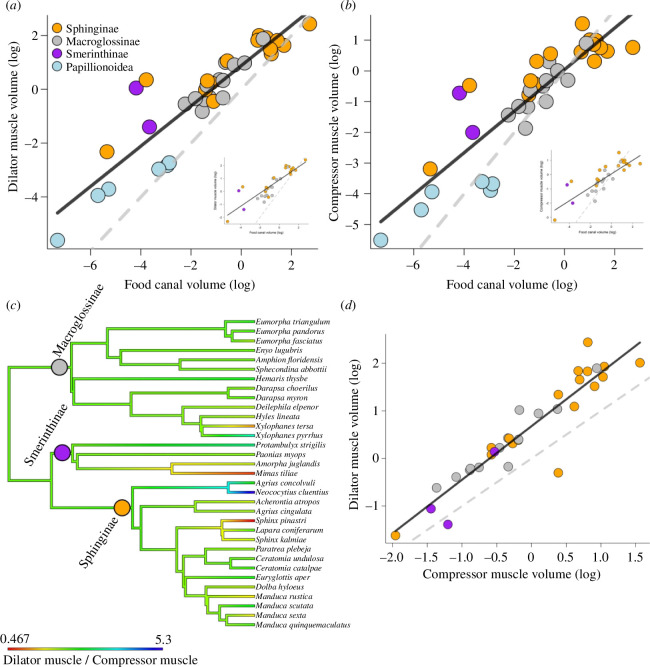
Evolution of muscles associated with the sucking pump in glossatan Lepidoptera. (*a*) Relationship between volume of the dilator muscles and food canal. Each colour-coded dot represents a species. Six species of butterflies (superfamily Papilionoidea) are represented in pale blue (from Julia Bauder and Harald Krenn, who provided additional data from [14]). The dashed grey line represents isometry (1 : 1 proportion); the solid line represents a standard regression with butterfly data. The inset represents the same data but excluding butterflies and using a phylogenetic regression model. (*b*) Same as (*a*) but representing compressor muscle volume. The inset represents the same data but excluding butterflies and using a phylogenetic regression model. (*c*) Ancestral reconstruction (based on the phylogeny of Kawahara & Barber [12]) of the ratio between the dilator muscle volume and compressor muscle volume. Branch lengths indicate timespan. Redder colours denote a larger compressor muscle when compared with the dilator muscle (ratio <1), and green–blue colours represent larger dilator muscle (ratio >1). Each dot on the phylogeny represents a subfamily of hawkmoths. Butterflies are phylogenetically distant from hawkmoths and were excluded from this analysis. (*d*) Same as (*a*) and (*b*) but showing the relationship between volume of the dilator muscles and compressor muscles.

When we consider only hawkmoths, the volume $V_{dm}$ of the dilator muscles increased with volume $V_{fc}$ of the food canal. The slope of the relationship was 0.5 (95% CI [0.4–0.6]), i.e. $V_{dm}\propto V_{fc}^{0.5}$ (figure 3*a*), and the strength of the correlation was high (phylogenetic generalized least-squares model (PGLS); adjusted *R*^2^= 0.76, *p* < 0.001), which is in line with existing models of drinking. When butterflies were added to the dataset, the slope increased to 0.75 (95% CI [0.6–0.9], i.e. $V_{dm}\propto V_{fc}^{0.75}$; figure 3*a*), and the correlation remained similar (ordinary least squares (OLS) regression; adjusted *R*^2^ = 0.8, *p* < 0.001).

The volume $V_{cm}$ of compressor muscles increased with volume $V_{fc}$ of the food canal (slope: 0.47, 95% CI [0.3−0.6], i.e. $V_{cm}\propto V_{fc}^{0.5}$; figure 3*b*). The strength of the correlation was also high (PGLS; adjusted *R*^2^ = 0.7, *p* < 0.001). When we added butterflies, the slope increased (slope: 0.67, 95% CI [0.5–0.8], i.e. $V_{cm}\propto V_{fc}^{0.67}$; figure 3*b*) as did the correlation (OLS regression; adjusted *R*^2^ = 0.8, *p* < 0.001). Based on the slopes, both regressions indicated that the amount of liquid in the food canal is equally relevant to the mechanical design of the muscles. Given that addition of butterflies does not change the trend, and the scaling patterns agree with predictions from our derivations (electronic supplementary material), we suggest that evolutionary pressures that shaped development of the sucking-pump complex might be similar throughout glossatan Lepidoptera.

To understand how selection might act on evolution of the sucking-pump muscles, we analysed the evolution of closely related species. We retrieved the rate of evolution of the muscles from the phylogenetic regression. We also performed another phylogenetic regression to test how evolutionarily coupled both muscles are. Since our goal was to test the evolutionary history of both muscles, we performed this analysis solely with hawkmoths. Further, we performed ancestral reconstructions of the ratio of the muscles for hawkmoths.

Evolutionary rates of the dilator and compressor muscles when regressed against food canal volume were similar (*σ*^2^ dilator: 0.01, 95% CI [0.008−0.02]; σ^2^ compressor: 0.05, 95% CI [0.02−0.65]), whereas both muscles were strongly coupled during the evolutionary history of Sphingidae (PGLS; slope = 1.03, 95% CI [0.92−1.14], $V_{dm}\propto V_{cm}^{1.03}$adjusted *R*² = 0.86, *p* < 0.0001; figure 2*d*). We also examined the raw data without phylogenetic correlations between species. The scaling was confirmed, $V_{dm}\propto V_{cm}^{1.04}$ (electronic supplementary material). Thus, changes in one muscle were mirrored by proportional changes in the other muscle while maintaining the magnitude in volume difference.

Hawkmoths differ in flight manoeuvres and foraging preferences [15,16], with some species not even feeding on nectar. These differences are mirrored in the evolutionary rate of development of dilator and compressor muscle volumes which changed from one species to another in a few million years (figure 3*c*). The correlation of the sucking-pump versus proboscis development in butterflies and hawkmoths suggests that hawkmoths might have similar biomechanical functionalities which were present when butterflies split from the common ancestor.

## 3. Flow can be neither continuous nor fully developed

The sucking pump of hawkmoths can accommodate approximately 1.2 times the food canal volume. Some butterflies have a similar ratio [11]. The sucking pump of monarch butterflies, *D. plexippus,* can accommodate about 1.4 times the food canal volume [11], which is well within the hawkmoth range.

**Figure 4.**
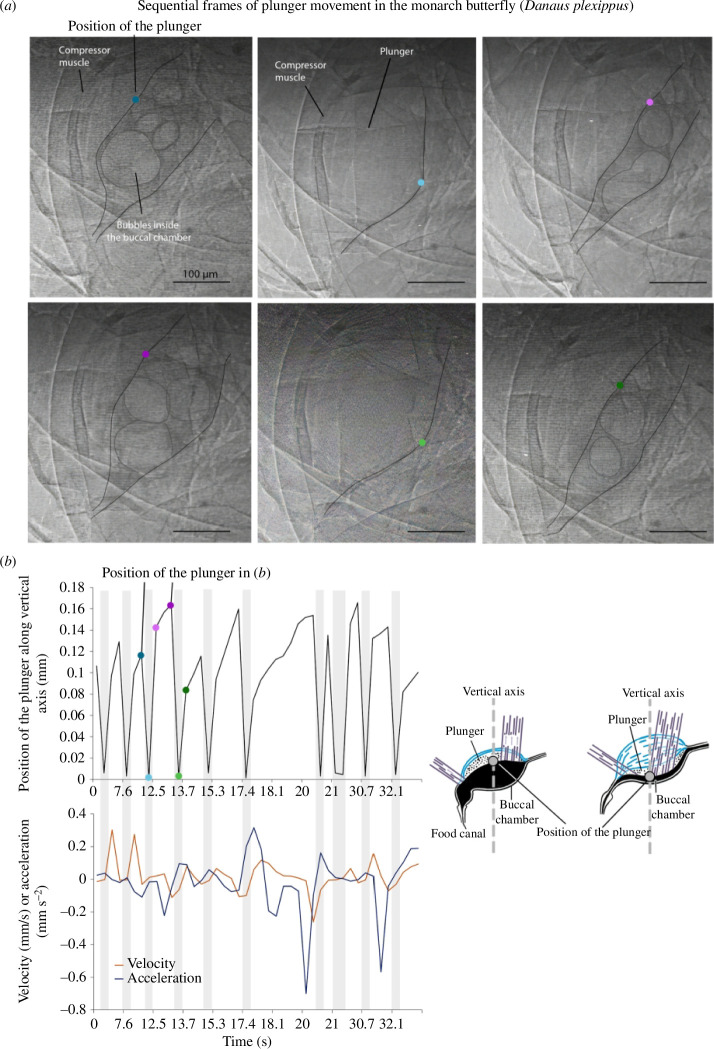
Flow speed is not constant during butterfly drinking. (*a*) Sequential frames of opening and closing of the plunger of the sucking pump of *Danaus plexippus*. Bubbles appear in the buccal chamber when the plunger rises. Scale bars represent 100 µm. Coloured dots represent the position of the plunger tracked in (*b*). (*b*) Tracking of the plunger along a vertical axis over time on the top graph, with velocity and acceleration on the lower graph. The black line represents the position of the plunger (grey dot). Coloured dots correspond to the plunger position in (*a*). Peaks correspond to when the plunger opens the chamber and valleys when the plunger closes it. Grey bars show timespans when the pump is closing.

Using X-ray phase-contrast videos of *D. plexippus* drinking (electronic supplementary material, video S1), we observed that flow through the food canal is pulsatile. We tracked the movement of the plunger (figure 4), showing that it continues to lift as liquid enters the sucking pump. The plunger then moves down and pushes liquid to the gut, leaving no room for liquid to move in (electronic supplementary material, video S1). The buccal chamber was not always full when the plunger closed, which is highlighted by the different peaks (figure 4*b*), suggesting that butterflies control their swallowing (figure 4*a,b*).

Velocity and acceleration of the plunger were not constant in *D. plexippus*, especially when the plunger was pulled, opening the lumen (electronic supplementary material, table S1). Velocity reached a maximum of 0.3 mm s^−1^ when the lumen was opened but given that the lumen can take several frames to open fully, mean velocity was low (0.06 mm s^−1^). Acceleration peaked when the pump was closing, reaching 0.7 mm s^−2^ (average = 0.1 mm s^−2^). This value is an underestimation because the plunger closed at a higher speed than the camera could register (30 fps); thus, the pump took less than 1/30 s to close.

The µCT measurements and X-ray imaging of butterfly drinking confirm that flow is not a steady stream of liquid through the proboscis to the buccal chamber. Each stroke is short; buccal chamber expansion takes about 1 s and contraction takes less than 1/30 s. The exponents of muscle volume versus food canal volume for the studied lepidopterans, $V_{dm}\propto V_{fc}^{0.75}$ and $V_{cm}\propto V_{fc}^{0.67}$, suggest that requirements for muscular power of dilators and compressors are similar. To investigate the biomechanical causes of this similarity, we evaluated flow features.

## 4. Bubble-train formation inside the food canal

**Figure 5.**
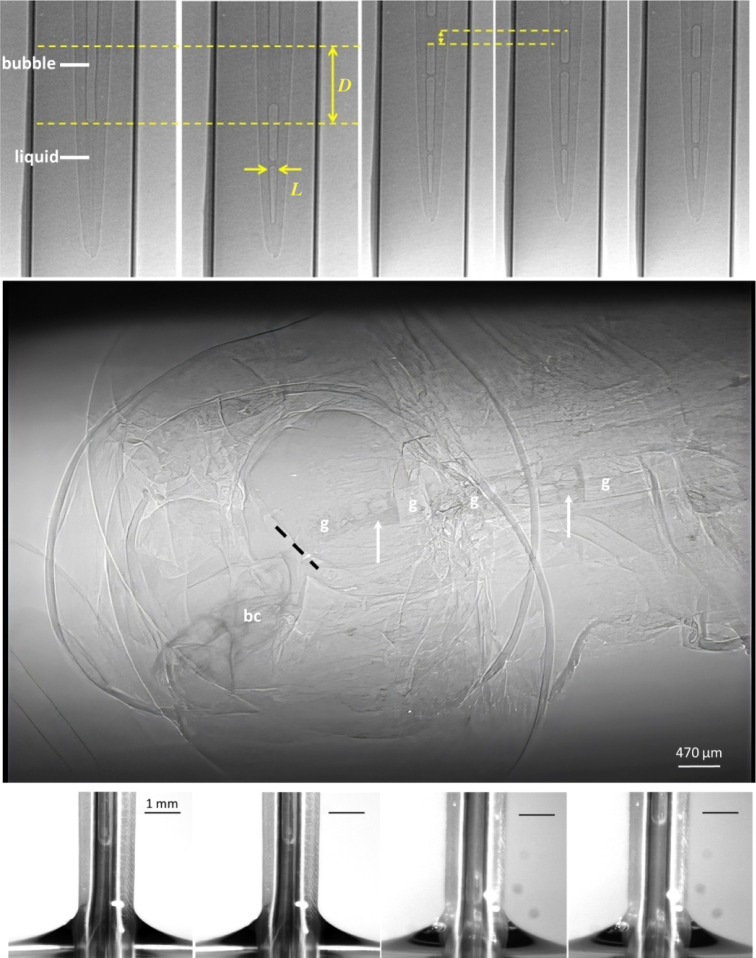
Bubble trains form when liquid enters the proboscis and are carried to the gut. X-ray imaging of (*a,b*) formation of an air bubble (brighter area) and secondary liquid bridge (darker area) in the proboscis of *Danaus plexippus*. The meniscus of the trapped drop in (*a*) becomes the frontal meniscus of the driving liquid bridge in (*b*). Distance is *D* = 571 μm and width of the bubble at its leading edge is *L* = 50 μm. (*c*–*e*) Generation of a bubble train and its movement from the tip toward the sucking pump at an average velocity of *V* = 0.273 mm s^−1^ calculated based on the displacement of the meniscus in (*c) *and (*d)* (yellow arrows). (*f*) Frame from X-ray phase-contrast video illustrating bubble-train movement from the buccal chamber (bc) to the gut (g). Dashed line indicates where the buccal chamber ends and the gut starts. Arrows indicate bubbles. (*g*–*j*) Images of *Manduca quinquemaculata* drinking. Bubbles form at the proboscis tip and at almost any position along the proboscis. Frames are separated by t= 4/25s.

X-ray imaging revealed bubbles in the food canal and the sucking pump of butterflies (figure 4; electronic supplementary material, video S1). Bubbles formed when a droplet broke up at the proboscis tip (figure 5*a–e*; electronic supplementary material, video S2). When butterflies applied suction pressure by raising the plunger (figure 4*a*), a drop broke up within tens of milliseconds. The camera frame rate was insufficient to detail break-up. Nonetheless, the tip of the proboscis is not airtight, and air could enter interlegular gaps when the butterfly creates negative pressure. Thus, a drop could break up when air from the legular bands merged into a bubble.

When the drop broke up, the driving liquid bridge moved towards the head (figure 5*a,b*). The time interval between frames (*a*) and (*b*) was *t* = 1/30 s; the meniscus in frame (*a*) moved a distance *D* = 571 μm, giving the average velocity 17.13 mm s^−1^. The corresponding Reynolds number, which measures the relative strength of inertial versus viscous forces, was Re=ρvR/η≈ 0.6, where *ρ* = 1000 kg m^−3^ is the water density, R≈35μm is the food canal radius and η=0.001 Pas is the water viscosity. This estimate suggests that during bubble formation, inertial and viscous forces were comparably important.

**Figure 6.**
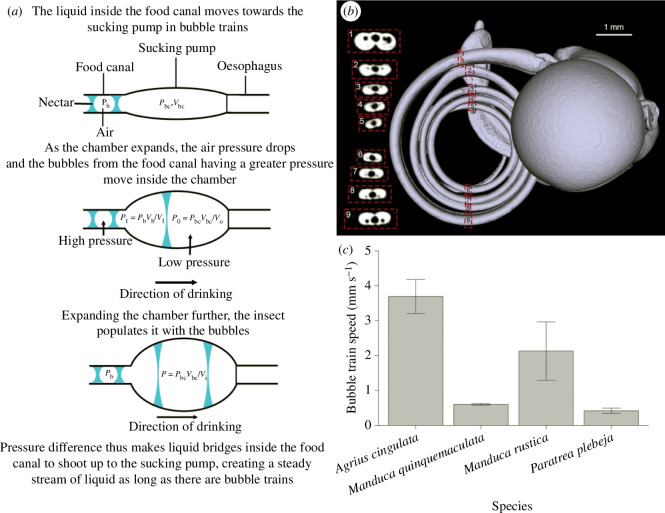
Filling the buccal chamber with bubbles. (*a*) When the plunger opens the chamber, the oesophagus remains closed. When the first liquid bridge arrives at the buccal chamber, air pressure and volume in the chamber are $P_{bc}$ and $V_{bc}$, respectively. The plunger keeps moving up, expanding the air volume and decreasing pressure in the chamber. When the pressure becomes lower than in the bubble, $P_{b}$, the first liquid bridge enters the chamber. The chamber is partitioned into two bubbles (volumes $V_{1}$ and $V_{0}$) with pressures $P_{1}=P_{b}V_{b}/V_{1}$ and $P_{0}=P_{bc}V_{bc}/V_{0}$, respectively. When pressures in adjacent bubbles are related as $P_{b}>P_{1}>P_{0}$, the second liquid bridge enters the chamber. Filling can continue when the plunger reaches its final height and the oesophagus opens. (*b*) The tapered food canal is smallest at the tip. (*c*) Mean bubble-train velocities in four species of hawkmoths; whiskers are standard deviations.

When the driving liquid bridge moved towards the sucking pump, it left a liquid film. When the bubble extended, the cylindrical surface on the side became unstable, and another, thinner liquid bridge was formed (figure 5*b*). The film on the food canal surface self-generated negative capillary pressure that kept pulling liquid from the outside. The process continued, and new liquid bridges formed (figure 5*c*,*d*). The butterfly partitioned the liquid into bubbles and drank the bubble train. The events in figure 5*a–e* happened within a single plunger rise (figure 4*a*), suggesting that energy of the dilator muscles was mostly spent on creating the first bubble. Secondary liquid bridges (i.e. the bubble train) formed spontaneously (figure 5*c–e*) at low Reynolds numbers owing to Plateau–Rayleigh instability [17,18] of the cylindrical film lining the wall of the food canal (figure 6*a*; electronic supplementary material; [7]).

Feeding experiments on four species of hawkmoths (*Manduca rustica*, *Manduca quinquemaculata*, *Agrius cingulata* and *P. plebeja*) confirmed that they swallow bubble trains [8]. The proboscis was uncoiled, and the flow features were observed at the meniscus (figure 5*g–j*; [8]). When hawkmoths drank, bubble trains moved up, allowing us to track flow speed inside the food canal.

Between drinking bouts, the same individual of *M. rustica* had an average bubble-train speed of 0.1 mm s^−1^ (maximum = 9.7 mm s^−1^). The pattern was similar for *P. plebeja*: the same individual in different repetitions had flow speeds of 0.1–0.8 mm s^−1^. In the same feeding bout, flow speed could vary: *A. cingulata* showed a maximum of 8.1 mm s^−1^ and a minimum 0.8 mm s^−1^ in one bout (figure 6*c*). These results confirm that drinking speed varies even within species and that longer proboscises do not necessarily slow fluid uptake (figure 6*c*).

We propose that partitioning liquid columns into bubble trains is the key biomechanical feature that allowed adaptation to feeding while hovering by increasing the speed of fluid uptake (figure 6*a*). The faster the bubble trains form, the faster the individual drinks and fills the sucking pump.

We assume that air in the bubbles follows the ideal gas law, and its pressure and volume are inversely proportional. The buccal chamber is not connected to the atmosphere; hence, the number of air molecules remains the same as the bubble expands. Therefore, the incoming bubble has greater pressure than the bubble inside the buccal chamber when the plunger rises, pushing the liquid bridge towards the chamber (figure 6*a*). The next bubble pushes its frontal liquid bridge to enter the chamber and the process repeats. Filling the buccal chamber may stop when pressure in bubbles of the incoming train equilibrates with pressure in bubbles collected in the buccal chamber. Opening the oesophagus and closing the cibarium allow bubbles in the buccal chamber to be swallowed when the dilators relax and the compressors contract, pulling the plunger to the pump floor.

Bubble trains in the tapered food canal (figure 6*b*) can be advantageous when no continuous column of liquid is formed. Bubble trains offer (i) self-propulsion of nectar bridges because bubbles closer to the tip have greater pressure than bubbles closer to the head, and (ii) drag reduction compared with a continuous liquid column, allowing drinking with minimal expenditure of muscular energy.

We estimate the corresponding drag reduction by the bubble train, using the Hagen–Poiseuille law (2.1). Consider a bubble of length $L_{b}$ in the bubble train with the liquid bridges of meniscus width W, so the number of bubbles is $N=L_{b}/W$ (i.e. one liquid bridge per bubble). To have the same velocity as the bubble train, a bubble-sized liquid column must experience a pressure differential of $L_{b}/W$ greater than the train per bubble (electronic supplementary material). $L_{b}/W$ > 10 in figure 5*f* suggests at least an order of magnitude reduction of viscous drag.

In the 100 µm diameter of the food canal, bubble velocities are in the order of millimetres per second (figure 6*c*). Such fast transport of liquids by vacuum pumps through 10 cm long microfluidic channels cannot be produced with continuous liquid columns [19].

In contrast to butterflies generating bubbles only at the hydrophilic drinking region near the proboscis tip [6], hawkmoths, with proboscises that are almost entirely hydrophilic, can form bubbles when liquid enters the dorsal legular band along their proboscises. How would bubble trains form in a long proboscis of hovering hawkmoths?

## 5. Bubble-train formation in hawkmoths

Hawkmoths can rapidly dip their hydrophilic proboscises into flowers while hovering, with nectar entering the proboscis through the legular bands (figure 6; [8]). The rate of nectar entering the food canal is controlled by permeability of these bands (on the order of 10^−15^ m^2^; [4,7]). We estimated that to fill the food canal through the legular bands submersed in nectar, hawkmoths would need less than 1 s, owing to capillary action [4]. This estimate agrees with observations on the hawkmoth *C. antaeus* spending less than 1 s at the orchid *D. lindenii* [3]. Therefore, a combination of capillarity-assisted absorption of nectar through the legular bands together with formation of bubbles at the free surface of the nectar-filled tube allows hawkmoths to rapidly transport nectar through the long proboscis to the sucking pump. This scenario is related to nectar uptake from a liquid pool.

When nectar is limited (e.g. to a film in the corolla), the fluid-uptake scenario must be modified because of the difficulties of forming a continuous liquid column. Field observations suggest that by moving the proboscis in the nectary back and forth, hawkmoths acquire a film on their proboscis [3]. Film thickness depends on proboscis size and withdrawal velocity. Given the large size and surface area of the proboscis, the nectar in this film cannot be ignored as a significant caloric reward.

**Figure 7.**
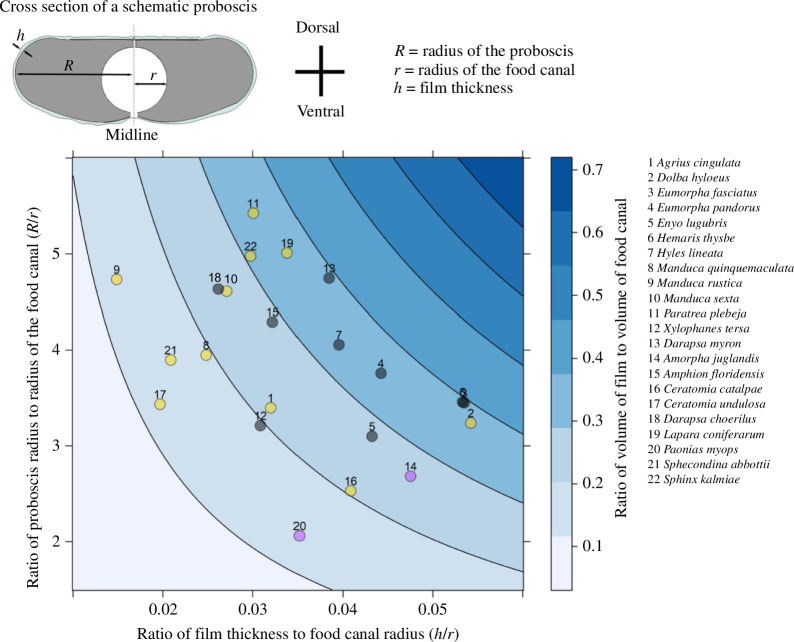
Characteristics of nectar films on hawkmoth proboscises after withdrawal from flowers. The lower the value on the colour scale, the smaller the liquid volume that the food canal could receive. The outermost distance from the symmetry axis of the proboscis cross-section is set to *R*, the radius of a model cylindrical proboscis; *r* is the radius of the food canal and *h* is thickness of the coating film on the proboscis. Yellow dots represent species of subfamily Sphinginae, grey dots the Macroglossinae and purple dots the Smerinthinae.


To determine if film volume is sufficient to generate bubble trains, we estimated film volume by modelling the proboscis as a circular cylinder of radius *R* (figure 7). This approximation allowed us to use equations 35–38 in [20] (electronic supplementary material) to measure thickness of the film around the proboscis when pulled from a flower. These equations require data on liquid viscosity, speed of cylinder withdrawal and cylinder geometry. We assumed that nectar viscosity is similar to that of water. We estimated speeds of proboscis withdrawal from our observations on hawkmoths in the wild; radii of the proboscises and food canals were estimated from µCT scans. We calculated film volume per unit length $V_{p}\approx 2\pi Rh,$ where h≪R is film thickness. We then related it to volume of the food canal per unit length $(V_{f}=\pi r^{2}$, where r is the food canal radius), using the equation

(5.1) $$\frac{V_{p}}{V_{f}}=2\left(\frac{h}{r}\right)\;\cdot \left(\frac{R}{r}\right).$$

The ratio $\frac{V_{p}}{V_{f}}$ ranged from 0.1 to 0.5 in all hawkmoth species (figure 7), i.e. nectar volume that remained on the proboscis surface was sizeable, enabling liquid bridges and bubble trains to form inside the food canal owing to Plateau–Rayleigh instability of cylindrical films. Bubble trains are expected to form naturally after the hawkmoth withdraws its proboscis from the flower.

## 6. Discussion

### (a) Morphological features of the sucking pump–proboscis complex reveal inconsistencies in existing models of glossatan feeding

We found evidence that current feeding models for Lepidoptera need reconsideration. Our µCT analysis revealed that hawkmoths may have a buccal chamber volume greater than the food canal volume. Thus, hawkmoths would be considered gulpers if they could swallow the entire volume of the food canal in a single sip. Pulling nectar from a 100 µm thin food canal would be akin to drinking honey through a straw. The insect would need strong dilator muscles. Yet, the dilator muscles are similar across species and feeding modes. The main difference is in muscle volume, which is a proxy for the capacity to accumulate mechanical energy: the greater the volume, the greater the potential energy for doing mechanical work.

Based on our calculations (electronic supplementary material), bubble trains provide an alternative solution to the problem in scenarios where suction pressure required to pull nectar through the food canal becomes greater than atmospheric pressure (when τ≪1, figure 2*b*). In this case, some bubbles should form, decreasing the energy required to pull liquid through the food canal.

If flow through the proboscis is not as important, fluid uptake and transport to the sucking pump can occur mainly through passive mechanisms. Morphological features of the proboscis [9] and behavioural features during feeding [6–8,21] indicate that fluid uptake is spontaneously driven by capillary action of the proboscis and surface tension of the fluid, where the radii of the food canal and galeae are the most important morphological parameters. Consequently, movements of the proboscis, such as antiparallel sliding and cracking [8,21], probably play a greater role in combatting viscous drag in the food canal than previously assumed.

### (b) Partitioning drops into bubble trains facilitates rapid fluid transport

Using X-ray phase-contrast and optical microscopies, we demonstrated that feeding can occur in pulses. Opening and closing the buccal chamber occur rapidly; to track the plunger, X-ray images must be taken in milliseconds (figure 4). No vacuum pump could circulate viscous fluids through micro-conduits at such a high rate.

X-ray and optical microscopies revealed bubble trains in the food canal and buccal chamber during drinking. Our numerical analysis suggests that proboscis geometry facilitates the formation of bubble trains because of the amount of nectar trapped on the outer surface of the proboscis in relation to the food canal.

The difference in feeding behaviour between landing lepidopterans and hovering hawkmoths is reflected in the mechanism of bubble-train formation. In butterflies, bubble trains are generated at the proboscis tip by ‘cavitating’ the trapped droplets. In contrast, hawkmoths can form bubble trains at any position along their hydrophilic proboscis, allowing them to drink from nectar pools and films coating the nectary walls.

Although the mechanism of generating bubble trains in landing and hovering lepidopterans differs, the strategy to break fluids into bubble trains to facilitate fluid transport is common. First, bubble trains provide an alternative drinking strategy when the amount of nectar is limited to a film on the inner surface of the corolla. They also minimize the force needed to pull and push the plunger, as viscous dissipation in the air is negligibly small, and the liquid bridges are self-propelled.

Second, dilator muscles of the sucking pump in glossatan Lepidoptera may not have the ability to generate a pressure differential of the magnitude necessary to drive a continuous liquid column at a sufficiently high velocity. Bubble-train viscosity in the Hagen–Poiseuille law is reduced by the ratio *W*/$L_{b}$ relative to the liquid column of the same length, $L_{b}$ (electronic supplementary material). Thus, to acquire small amounts of nectar when dipping, the bubble-train mechanism becomes preferential, and the shorter the bubble train, the faster the drinking. For example, by forming shorter bubble trains, the hawkmoth could sip at lower pressure differentials (electronic supplementary material).

In early lineage Lepidoptera that cannot link the galeae, their C-shaped, hairy galeae would generate strong capillary suction to pull fluid towards the sucking pump. Therefore, bubble trains would not have been needed, as fluid would flow passively towards the sucking pump. Once Glossata evolved the ability to unite the galeae, the bubble-train mechanism could have facilitated diversification, enabling evolution of Glossata with increasingly longer proboscises, without significant changes in musculature and sucking pump–proboscis features. Production of bubble trains translates to faster drinking, which would reduce exposure to predation, reduce desiccation risk by enhancing the ability to acquire minute quantities of limited nectar, and increase floral sampling rate. Thus, in addition to the ability to drink a continuous column of fluid from pools, the ability to take advantage of fluid films in floral tubes and on other nutrient sources (e.g. sap flows and damp soil) by capitalizing on bubble trains would have enhanced the range of food sources, setting the scene for diversification of Lepidoptera.

### (c) The proboscis as the main target of evolutionary pressure and the path for Sphingidae diversification

The connection between morphology and function provides the landscape for selection to act. Any positive selection on function should cascade down to morphological characteristics that can improve a given function, and *vice versa*. This is a basis for morphological diversification, whereby characteristics more associated with changes in function should change faster than characteristics less associated with changes in function. Therefore, if bubble-train transport is a low energy-consuming feeding mechanism, changes in proboscis morphology towards transport optimization should influence feeding performance more than changes in the sucking pump. These changes in proboscis morphology would allow hawkmoths to drink from variable nectar sources, from pools to films. The moths could quickly dip the proboscis into a floral tube, collect nectar on the proboscis surface and transform the film into bubble trains for uptake. Evolutionary changes in this direction would decrease the necessity of staying longer at the same flower to drink. Our results, and numerous studies of the evolution of proboscis length, converge on this point: proboscises are evolutionarily labile.

Several features of the proboscis indicate high rates of evolution, whereas the sucking pump is more static. This relationship holds for proboscis length, the most well studied characteristic of Sphingidae [22], but if we consider diversity as a proxy for rate of evolution, other proboscis characteristics also show high rates of evolution, even though they have not been measured with modern phylogenetic comparative methods. For instance, the tip of the proboscis varies across Lepidoptera, with some species showing minimal differentiation and others showing elaborate brushy tips [23]. The tapering angle of the proboscis, radii of curvature and wettability are also largely variable across Sphingidae [8].

Both compressors and dilators of the sucking pump, however, have low evolutionary rates. Our modelling shows that exchanging the sucking pump of a honey-drinking species (e.g. *Acherontia atropos*) for the sucking pump of a nectar-drinking species would still allow the former species to drink honey [11]. Therefore, owing to the high rates of evolution, all features that characterize the proboscis are targets of selection when feeding performance is concerned. The path to diversification of the Sphingidae—and possibly most glossatan Lepidoptera—has thus gone through the proboscis.

## 7. Methods

### (a) Species collection and micro-computed tomography

We used hand nets and UV lights to collect hawkmoths in Clemson, SC, USA, in the summers of 2021 and 2022. We sampled at least one individual of 20 species. Two additional species, *M. sexta* and *C. catalpae*, were purchased from biological supply companies and laboratory-reared from larvae and pupae, respectively. We also used the biometric data of 11 species in table 4 of [24]. All measurements are in electronic supplementary material, table S2.

We put the hawkmoths in an ultra-low freezer (−76°C) to preserve conformation of soft tissues. The frozen hawkmoths were shipped on ice overnight to North Dakota State University Electron Microscopy Core Lab for µCT. Scanning was conducted within 2 days after receipt of the specimens (electronic supplementary material). We imaged one individual from each of the 22 species. If the sucking pump of a specimen collapsed during scanning, we scanned another individual. To determine if our procedure influenced measurements, we scanned two individuals of *Enyo lugubris* and two of *A. cingulata*. The scans showed little variation (electronic supplementary material, figure S2A,B and figure S3).

### (b) Measurements of sucking-pump characteristics

To investigate the evolutionary correlation between structures, we used a multigene, time-calibrated molecular phylogeny of Sphingidae [12]. We pruned the tree for our species. Ten of 33 species were not in the tree. Eight species were replaced by other species of the same genus, and two were manually added to the phylogeny (electronic supplementary material).

To test the strength of the correlation between dilator muscles and food canal volume, we used a PGLS implemented in the package *phylolm* [25]. Given two species, *C. catalpae* and *M. tiliae*, did not have a united proboscis, we removed them from any analyses that required food canal volume. We used a PGLS model because it allowed the alternative models of evolution, while other phylogenetic models typically only allow Brownian motion. We used dilator muscle volume as the dependent variable and food canal volume as the independent variable. We modelled trait evolution using Brownian motion and Ornstein–Uhlenbeck models and added an error measurement associated with sucking-pump volume. The best model had the lowest Akaike’s information criterion (AIC), with more than two units of AIC difference from the other models (electronic supplementary material, table S3). All models were run with a bootstrap of 10 000 replications to calculate confidence intervals. We applied a similar model using compressor, instead of dilator, muscle volume as the dependent variable. All variables were log-transformed to linearize any underlying geometrical relationship between variables [26].

To test how addition of butterflies could change the relationship between muscles and the food canal, we obtained data from six species of butterflies (family Riodionidae): *Eurybia lycisca*, *Eurybia livina*, *Eurybia unxia*, *Sarotia gyas*, *Eusalia aurantia* and *Mesosemia asa* [14]. The authors in [14] scanned two individuals per species, so we averaged the values and used them in our analysis. To test the correlation between muscles and the food canal, we used the procedure above but with OLS regression rather than phylogenetic regression. No available phylogeny contains both the hawkmoth and butterfly species but, because these groups are sufficiently distant from one another, a phylogeny would not reveal a meaningful pattern without sampling species between these groups. We conducted all statistical analyses in R software [27].

We used a PGLS model to test the strength of the evolutionary correlation between dilator and compressor muscles. Our procedure was similar to that outlined above for food canal volume. Our second approach used an ancestral reconstruction to visualize how the ratio between both muscles changed through the phylogeny. To do this, we used the function *contMap* from the *phytools* package [28].

### (c) Bubble-train imaging at Argonne National Laboratory

Filming of monarch butterfly feeding was done at the Advanced Photon Source of Argonne National Laboratory. All details on filming are in [7] and the electronic supplementary material.

### (d) Characterization of flow dynamics during hawkmoth feeding

In the drinking experiments, we uncoiled the proboscis of live hawkmoths and inserted the tip in a tube inside a beaker with deionized water. We filmed hawkmoths drinking at 30 fps (Allied Vision Stingray camera) with lighting from a 60 W halogen source. To understand how the radius of the food canal influences flow dynamics, we performed the experiment for four species that differed in the radius of their food canal: *M. sexta* (*n* = 3), *M. quinquemaculata* (*n* = 5), *A. cingulata* (*n* = 3) and *P. plebeja* (*n* = 4).

### (e) Modelling nectar deposition on the proboscis

We filmed 13 feeding bouts of *M. sexta* and *M. rustica* in the natural environment and calculated the time each individual took to remove its proboscis from flowers of *Hedychium coronarium*. On average, individuals removed the proboscis in 0.5 s. For species with approximately 10 cm long proboscises, the speed of withdrawal was estimated as 0.2 m s^−1^. We used this speed for all species and used µCT scans to measure cross-sectional diameter for at least five points along the proboscis and then averaged all measurements to obtain the average radius. These measurements allowed us to calculate thickness of the film on the proboscis surface, using mathematical formulations in [20], and evaluate equation (5.1).

## Data Availability

All relevant data used for analysis to support this paper are available from Dryad [29]. Supplementary material is available online [30].
